# Cryptochrome magnetoreception: four tryptophans could be better than three

**DOI:** 10.1098/rsif.2021.0601

**Published:** 2021-11-10

**Authors:** Siu Ying Wong, Yujing Wei, Henrik Mouritsen, Ilia A. Solov'yov, P. J. Hore

**Affiliations:** ^1^ Institut für Physik, Carl-von-Ossietzky Universität Oldenburg, Oldenburg 26111, Germany; ^2^ Department of Chemistry, University of Oxford, Oxford OX1 3QZ, UK; ^3^ Institut für Biologie und Umweltwissenschaften, Carl-von-Ossietzky Universität Oldenburg, Oldenburg 26111, Germany; ^4^ Research Centre for Neurosensory Science, University of Oldenburg, Oldenburg 26111, Germany

**Keywords:** magnetoreception, cryptochrome, radical pairs, magnetic field effects, migratory songbirds

## Abstract

The biophysical mechanism of the magnetic compass sensor in migratory songbirds is thought to involve photo-induced radical pairs formed in cryptochrome (Cry) flavoproteins located in photoreceptor cells in the eyes. In Cry4a—the most likely of the six known avian Crys to have a magnetic sensing function—four radical pair states are formed sequentially by the stepwise transfer of an electron along a chain of four tryptophan residues to the photo-excited flavin. In purified Cry4a from the migratory European robin, the third of these flavin–tryptophan radical pairs is more magnetically sensitive than the fourth, consistent with the smaller separation of the radicals in the former. Here, we explore the idea that these two radical pair states of Cry4a could exist in rapid dynamic equilibrium such that the key magnetic and kinetic properties are weighted averages. Spin dynamics simulations suggest that the third radical pair is largely responsible for magnetic sensing while the fourth may be better placed to initiate magnetic signalling particularly if the terminal tryptophan radical can be reduced by a nearby tyrosine. Such an arrangement could have allowed independent optimization of the essential sensing and signalling functions of the protein. It might also rationalize why avian Cry4a has four tryptophans while Crys from plants have only three.

## 1. Introduction

The remarkable magnetic compass sense that helps night-migratory songbirds navigate thousands of kilometres [1,2] is thought to have a photochemical mechanism [3–8]. The axial nature [1,9] and the light-dependence [10] of the birds’ responses to the geomagnetic field, together with the involvement of the birds’ visual system in processing magnetic compass information [11,12], are consistent with the formation of transient, magnetically sensitive radical pairs in photoreceptor cells in the retina [5]. The molecule that plays host to this photochemistry seems likely to be a member of the cryptochrome (Cry) family of proteins [13–15], a possibility first suggested more than 20 years ago [3]. Of the six known avian Crys [14,16–25], Cry1a and Cry4a are the main contenders (reviewed in [6,7]). There is also debate about whether the magnetically sensitive radical pairs are formed directly by photo-excitation of the protein or indirectly as intermediates during ‘dark’ back-reactions [26–32]. The identity of any ‘dark’ radical pair is unknown and vertebrate Cry1a does not seem to bind the crucial flavin adenine dinucleotide (FAD) chromophore at all strongly *in vitro* [33]. We focus here on Cry4a in which flavin–tryptophan radical pairs [34–38] arise from a series of electron transfers along a chain of aromatic amino acid residues that stretches approximately 25 Å from the FAD in the interior of the protein out to its surface [23,39]. In contrast to plant Crys, in which three tryptophans (Trp), or two tryptophans and a tyrosine (Tyr), constitute the electron transfer pathway [13], animal and animal-like Crys possess a tetrad of tryptophans (e.g. avian Cry4s [23,39]) or three tryptophans plus a terminal tyrosine (e.g. *Chlamydomonas reinhardtii* Cry [40]).

**Figure 1.**
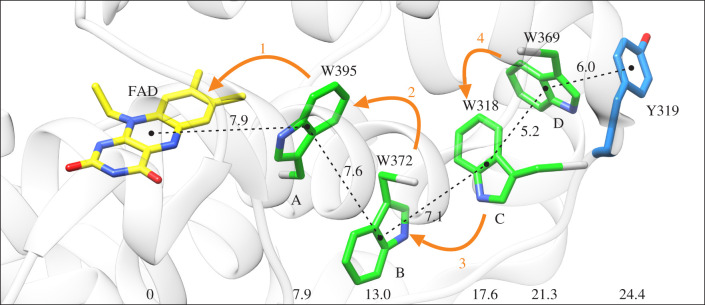
Structure of the electron transfer chain in *Cl*Cry4a (PDB: 6PU0 [37]) showing the FAD chromophore, the tryptophan tetrad and Tyr319. The numbers between adjacent groups are centre-to-centre separations in Å. The numbers at the bottom of the figure are centre-to-centre distances from the FAD. The orange arrows indicate the four sequential electron transfers. Only the isoalloxazine part of the FAD is shown.

Figure 1 shows the structure of the flavin component of the FAD and the four tryptophans (W) in pigeon (*Columba livia*, *Cl*) Cry4a, labelled: A (W395), B (W372), C (W318) and D (W369) [37]. The sequence numbers are the same for Cry4a from the night-migratory European robin (*Erithacus rubecula*, *Er*) [21]. Also shown is the sidechain of Tyr319, positioned at the far end of the Trp-tetrad, in contact with solvent. Photo-excitation of FAD in *Er*Cry4a is followed by four consecutive electron transfers between adjacent donors/acceptors, producing four sequential radical pairs: [FAD^•−^ Trp_X_H^•+^] (abbreviated RP1*_*X*_*, X = A, B, C or D) [39]. The separations of the flavin and tryptophan radicals in these four states of *Cl*Cry4a are approximately 8, 13, 18 and 21 Å, respectively [37]. Judging by molecular dynamics simulations, the electron transfer chain in *Er*Cry4a has a very similar structure with very similar distances between the key components [39].

A recent study of purified robin Cry4a by Xu *et al*. [39], the first of its kind for any migratory animal Cry, has shed considerable light on the performance of the protein as a potential magnetoreceptor. Spectroscopic measurements were made on the wild-type (WT) protein, and four mutants, W_X_F (X = A, B, C or D), in which each of the four tryptophans, in turn, had been replaced with phenylalanine (F) to block electron transfer at different points along the chain. The main findings are as follows. (i) Unlike some other avian Crys [33], *Er*Cry4a can be purified with the essential FAD chromophore stoichiometrically bound. (ii) Blue-light irradiation of W_A_F, W_B_F and W_C_F mutants either yielded no detectable radicals (W_A_F) or produced FAD and Trp radicals that are too short-lived to be magnetically sensitive (W_B_F and W_C_F). In the W_D_F mutant and the WT protein, however, light-induced radicals with lifetimes in excess of 100 ns were identified. (iii) From measurements of the radical–radical separations, it is clear that RP1_D_ is the dominant transient charge-separated state in the WT protein. (iv) Smaller magnetic field effects were found for purified WT *Er*Cry4a than for two proteins with only three tryptophans: the W_D_F mutant of *Er*Cry4a and Cry1 from the model plant, *Arabidopsis thaliana* (*At*Cry1) [39].

If Cry4a is the magnetic sensory molecule in migratory songbirds and if, *in vivo*, the RP1_C_ state is more magnetically sensitive than RP1_D_ (as is the case *in vitro*), then one might wonder why Cry4a has a Trp-tetrad instead of a Trp-triad. This is the question we address here. We explore the proposal [39] that, under the right conditions, a Trp-tetrad would be consistent with high detection sensitivity *and* might have allowed independent evolutionary optimization of the two essential functions of the protein—sensing and signalling. The key assumption underlying this idea is that RP1_C_ and RP1_D_ interconvert by fast reversible electron hopping, i.e. ${\mathrm{FAD}}^{∙-}{\mathrm{Trp}}_{C}H^{∙+}{\mathrm{Trp}}_{D}H↔{\mathrm{FAD}}^{∙-}{\mathrm{Trp}}_{C}{H\;\mathrm{Trp}}_{D}H^{∙+}$ [39].

## 2. Radical pair reaction schemes

**Figure 2.**
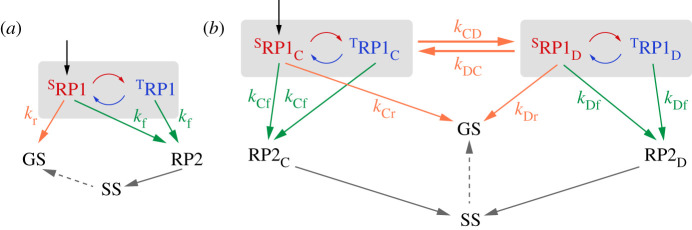
(*a*) Conventional reaction scheme in which magnetic field effects on the yield of the signalling state (SS) come from a single radical pair, RP1 (=RP1_C_ or RP1_D_) [41]. Singlet and triplet states are labelled S and T, respectively. (*b*) Modified reaction scheme in which RP1_C_ and RP1_D_ both contribute to the magnetic field effect [39]. The vertical black arrows indicate the formation of singlet radical pairs by spin-conserving electron transfer along the Trp-triad or tetrad to the photo-excited singlet state of FAD. Orange arrows: electron transfer reactions. Green arrows: TrpH^•+^ deprotonation reactions. Curved red/blue arrows: coherent singlet–triplet interconversion. The symbols beside the arrows are rate constants. GS denotes the ground state of the protein.

Figure 2*a* shows part of the conventional Cry reaction scheme in the case that the magnetic field effect stems from a single radical pair (RP1 = RP1_C_ or RP1_D_) [41]. RP1 is formed by electron transfer along the tryptophan triad or tetrad to the photo-excited FAD (not shown) and interconverts coherently between its singlet (^S^RP1) and triplet (^T^RP1) states. At the same time, ^S^RP1 returns to the ground state (GS) by spin-selective back electron transfer (rate constant *k*_r_; r = recombination reaction) while both ^S^RP1 and ^T^RP1 can proceed to a stabilized radical pair state, RP2 (rate constant *k*_f_; f = forward reaction) [41]. In the latter step, a proton is lost from the indole nitrogen of the tryptophan radical, TrpH^•+^ → Trp^•^, to produce either [FAD^•−^ Trp_C_^•^] (RP2_C_) or [FAD^•−^ Trp_D_^•^] (RP2_D_). The magnetic field effect manifests as a change in the yield of RP2 and hence that of the signalling state (SS), a more stable form of the protein in which we assume the tryptophan radical has been reduced (Trp^•^ → TrpH) and the flavin radical protonated (FAD^•−^ → FADH^•^). SS then returns to the GS of the protein on a much longer timescale. We assume RP2 lives long enough *in vivo* (more than 10 µs) that its electron spins are fully relaxed before conversion to SS so that it generates no additional magnetic field effects. In the simulations described below, we calculate the dependence of the quantum yield of SS (*Φ*_SS_, assumed to equal that of RP2) on the direction of the external magnetic field with respect to an array of mutually aligned Cry molecules. This quantity represents the ‘signal’ from which a bird could derive a magnetic compass bearing. Conversion of GS to SS is thought to lead to a conformational change that alters the protein's binding affinity to signalling partners and thereby initiates a biochemical signalling cascade [42].

**Figure 3.**
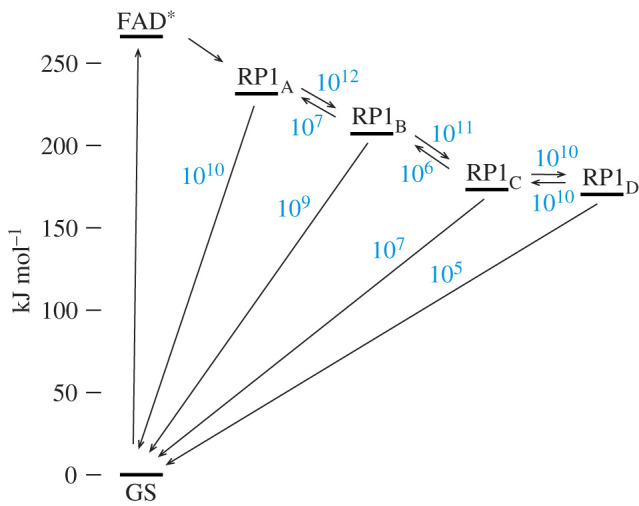
Approximate rate constants (in s^−1^) for electron transfer reactions in *Er*Cry4a. Values for RP1_A_ → GS and RP1_B_ → GS come from transient absorption experiments [39]. All other rate constants, and the free energies of the various states come from molecular dynamics simulations [39]. FAD* is the photo-excited singlet state of FAD. The RP2 and SS states are not shown.

The notion that RP1_C_ and RP1_D_ might jointly be responsible for the magnetic sensitivity of *Er*Cry4a came from estimates of electron transfer rate constants (figure 3) derived from molecular dynamics simulations [39]. The first two steps along the chain of four tryptophans (RP1_A_ → RP1_B_ and RP1_B_ → RP1_C_) were found to be rapid, exergonic and essentially irreversible. At each stage, forward electron transfer is two orders of magnitude faster than direct return to the GS, such that the RP1_C_ state would be formed in high yield. By contrast, RP1_C_ and RP1_D_ were found to have free energies differing by approximately *k*_B_*T* at physiological temperatures, with similar forward (*k*_CD_) and backward (*k*_DC_) electron transfer rate constants for the interconversion of the two states. The estimates of *k*_CD_ and *k*_DC_ (approx. 10^10^ s^−1^ [39]), are considerably faster than both the singlet–triplet interconversion and the subsequent reactions of RP1_C_ and RP1_D_, implying that both radical pairs may contribute to magnetic sensing. We, therefore, explore a modified reaction scheme, figure 2*b*, involving the two interconverting radical pairs in which one electron spin is on the flavin and the other resides on either Trp_C_H or Trp_D_H [39]. The singlet states of both pairs can return to the GS (rate constants *k*_Cr_ and *k*_Dr_) and the TrpH^•+^ radicals can be deprotonated to form the RP2_C_ and RP2_D_ states (rate constants *k*_Cf_ and *k*_Df_), which then proceed to the SS, again assumed to contain FADH^•^ as the only radical. In this modified reaction scheme, the yield of the SS, *Φ*_SS_, remains the quantity of interest and is defined as the sum of the yields of RP2_C_ and RP2_D_.

## 3. Methods

**Figure 4.**
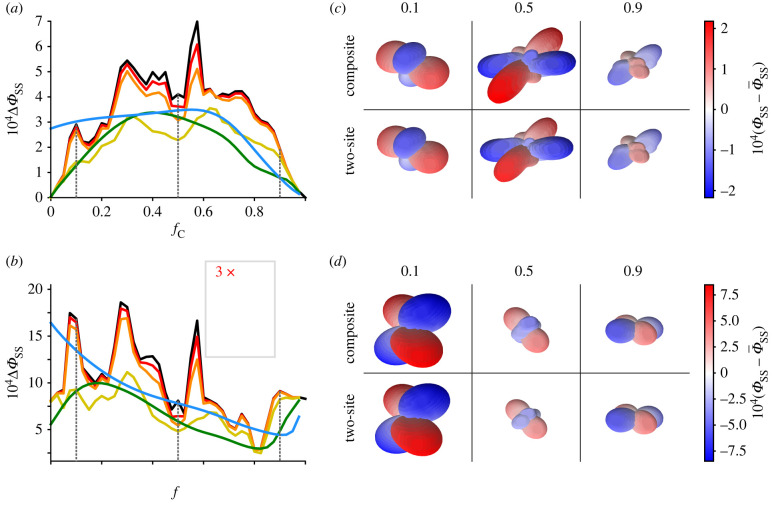
Comparisons of the anisotropy of the SS quantum yield, Δ*Φ*_SS_, for the two-site and composite radical pair models. (*a*,*b*) dependence of Δ*Φ*_SS_ on *f*_C_ with *k*_CD_ values in s^−1^ as indicated. (*c*,*d*) Anisotropy surface plots for *f*_C_ = 0.1, 0.5 and 0.9 (corresponding to the vertical dashed lines in (*a*) and (*b*)) for the composite and two-site models with *k*_CD_ = 3 × 10^10^ s^−1^. (*a*,*c*) *k*_Cr_ = *k*_Df_ = 10^6^ s^−1^, *k*_Dr_ = *k*_Cf_ = 0. (*b*,*d*) *k*_Cr_ = 1.2 × 10^7^ s^−1^, *k*_Dr_ = 3.4 × 10^5^ s^−1^, *k*_Cf_ = *k*_Df_ = 10^6^ s^−1^. The spin-relaxation rate was *k*_relax_ = 10^6^ s^−1^ in all cases. In (*a*) and (*b*) the black trace, obtained using the composite model, corresponds to the two-site model in the limit of infinitely fast electron hopping. The anisotropy surface plots (*c*) and (*d*) were obtained by calculating *Φ*_SS_(*Ω*) for 1601 spherically distributed magnetic field directions, *Ω*. The distance from the centre of the plot to the surface in a direction *Ω* is proportional to the difference between *Φ*_SS_(*Ω*) and the spherical average, $\bar{\Phi }$_SS_. Red and blue indicate magnetic field directions in which *Φ*_SS_(*Ω*) is larger and smaller, respectively, than $\bar{\Phi }$_SS_. See electronic supplementary material, section S3 for additional anisotropy surface plots.

**Figure 5.**
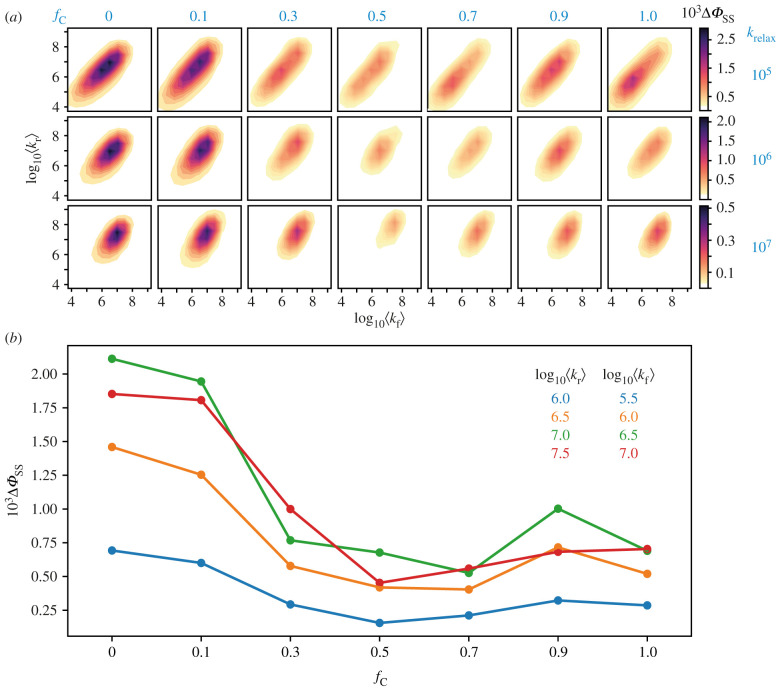
(*a*) Dependence of Δ*Φ*_SS_ on log_10_$\left\langle k_{f}\right\rangle$and log_10_$\left\langle k_{r}\right\rangle$, calculated using the composite model for seven values of *f*_C_ (columns) and three values of *k*_relax_ (10^5^, 10^6^, 10^7^ s^−1^, rows). The nuclear spins included in the calculation were FN5, WN1 and WH1. (*b*) Variation of Δ*Φ*_SS_ with *f*_C_, for four combinations of $\left\langle k_{r}\right\rangle$ and $\left\langle k_{f}\right\rangle$, with *k*_relax_ = 10^6^ s^−1^. The data shown in (*b*) were taken from the middle row of (*a*). The values of $\left\langle k_{r}\right\rangle$ and $\left\langle k_{f}\right\rangle$ for (*b*) were chosen to satisfy the condition for Δ*Φ*_SS_ to be large, i.e. $\left\langle k_{r}\right\rangle \approx 3\left\langle k_{f}\right\rangle$ and 3 × 10^5^ s^−1^ ≤ *k*_f_ ≤ 10^7^ s^−1^.

The reactions shown in figure 2*b* were modelled by means of coupled stochastic Liouville equations, one for each of the two states, RP1_C_ and RP1_D_ (see electronic supplementary material, section S1 for details). The spin Hamiltonians of the two radical pairs comprised electron Zeeman, electron–nuclear hyperfine and electron–electron dipolar interactions. Haberkorn operators were used for the recombination (*k*_Cr_ and *k*_Dr_) and forward (*k*_Cf_ and *k*_Df_) reaction steps [43]. Dipolar tensors [44] were calculated using the centre-to-centre vectors for FAD–Trp_C_ and FAD–Trp_D_ in *Cl*Cry4a [37]. The intensity of the geomagnetic field was 50 µT in all simulations. The anisotropy of the quantum yield of the SS,

3.1 $$\Delta \Phi_{\mathrm{SS}}=\max(\Phi_{\mathrm{SS}})-\min(\Phi_{\mathrm{SS}}),$$

was calculated as a measure of the magnetic compass sensitivity, where *Φ*_SS_ is the sum of the yields of RP2_C_ and RP2_D_. The maximum and minimum values of *Φ*_SS_ were determined by sampling, respectively, 1601 (figure 4) and 98 (figure 5) spherically distributed magnetic field directions. Note that this Δ*Φ*_SS_ differs from the quantity plotted in figure 4*c* of Xu *et al*. [39] which is the change in the isotropic yield of the SS induced by a 50 µT magnetic field, calculated using the reaction scheme in figure 2*a*.

Electron spin relaxation, with rate constant *k*_relax_, was included by modelling the effects of isotropic, randomly fluctuating local magnetic fields [45] (electronic supplementary material, equation S7). The rate of spin relaxation of radical pairs in Cry has not been determined experimentally. The best estimates of *k*_relax_ come from a study of Cry1 from the plant *A. thaliana*, using a combination of all-atom molecular dynamics simulations and Bloch–Redfield relaxation theory [45,46]. Librational motions of the FAD^•−^ and TrpH^•+^ radicals and fluctuations in their positions and dihedral angles modulate hyperfine and dipolar interactions and thereby induce spin relaxation at rates in the range 10^6^–10^7^ s^−1^. The simulations described below were performed with *k*_relax_ = 10^6^ s^−1^ (figure 4) and *k*_relax_ = 10^5^, 10^6^ or 10^7^ s^−1^ (figure 5). Values of *k*_relax_ ≈ 10^6^ s^−1^ allow time for electron Larmor precession (frequency = 1.4 MHz in a 50 µT field) to affect the spin dynamics before the spin coherence is irreversibly lost.

If RP1_C_ and RP1_D_ interconvert sufficiently rapidly, we anticipate that they can be treated as a single ‘composite’ radical pair described by figure 2*a* with a single stochastic Liouville equation (see electronic supplementary material, section S1 for details). The hyperfine and dipolar interactions of this composite species are averages, weighted by the fractional equilibrium populations of RP1_C_ and RP1_D_:

3.2 $$f_{C}=\frac{k_{\mathrm{DC}}}{k_{\mathrm{CD}}+k_{\mathrm{DC}}}\;\mathrm{and}\;f_{D}=\frac{k_{\mathrm{CD}}}{k_{\mathrm{CD}}+k_{\mathrm{DC}}}.$$

Weighted-average rate constants were obtained similarly:

3.3 $$\left\langle k_{r}\right\rangle =f_{C}k_{\mathrm{Cr}}+f_{D}k_{\mathrm{Dr}}\;\mathrm{and}\;\left\langle k_{f}\right\rangle =f_{C}k_{\mathrm{Cf}}+f_{D}k_{\mathrm{Df}}.$$

Hyperfine tensors, calculated by density functional methods [47], were rotated to match the relative orientations of FAD, Trp_C_ and Trp_D_ in the crystal structure of *Cl*Cry4a [37,48]. As the computational resources required for the simulations scale steeply with the number of spins, only a subset of the hyperfine interactions in the flavin and tryptophan radicals could be included. From among the nuclei with the largest anisotropic hyperfine interactions, three were selected (see electronic supplementary material, figure S1 for atom labelling schemes): N5 in FAD^•−^ (FN5) and N1 and H1 in each of Trp_C_H^•+^ and Trp_D_H^•+^ (WN1, WH1). FN5 and WN1 were used for the calculations shown in figure 4. All three nuclear spins were used for figure 5. The Liouvillian matrices for the two-site and composite models had dimensions 32*Z*^2^ and 16*Z*^2^, respectively, where *Z* = 27 or 108 for the two- and three nuclei calculations, respectively.

## 4. Results

We start by comparing the two-site RP1_C_ ↔ RP1_D_ approach (figure 2*b*) with the composite model (figure 2*a*) in which the two rapidly interconverting radical pairs act as a single entity with weighted-average properties. Figure 4 shows the dependence of the reaction yield anisotropy, Δ*Φ*_SS_, on the rate constants (*k*_CD_ and *k*_DC_) for interconversion of RP1_C_ and RP1_D_ for two sets of recombination and forward reaction rate constants (*k*_Cr_, *k*_Cf_, *k*_Dr_ and *k*_Df_). The first set, used for figure 4*a*,*c*, corresponds to the extreme case in which recombination is exclusively from RP1_C_ and the forward reaction is exclusively that of RP1_D_: *k*_Cr_ = *k*_Df_ = 1.0 × 10^6^ s^−1^, *k*_Dr_ = *k*_Cf_ = 0. In the second set, used for figure 4*b*,*d*, *k*_Cr_ = 1.2 × 10^7^ s^−1^ and *k*_Dr_ = 3.4 × 10^5^ s^−1^ (estimates from Xu *et al*. [39]) and *k*_Cf_ = *k*_Df_ = 1.0 × 10^6^ s^−1^. For both sets, the 1.0 × 10^6^ s^−1^ values were chosen (i) to allow time for the 50 µT magnetic field to significantly affect the spin dynamics and (ii) so that the forward reaction can compete with recombination.

In figure 4*a*,*b*, Δ*Φ*_SS_ is plotted (in colour) as a function of *f*_C_, the fraction of radical pairs in the RP1_C_ state, for five values of *k*_CD_, with *k*_DC_ given by *f*_C_*k*_CD_/(1 − *f*_C_) (equation (3.2)). Also shown are the equivalent calculations for the composite radical pair (in black). As anticipated, the correspondence between the two-site and composite models improves as *k*_CD_ and *k*_DC_ are increased, with respectable, albeit not perfect, agreement when *k*_CD_ > 10^10^ s^−1^, a condition satisfied by the *k*_CD_ and *k*_DC_ values estimated by Xu *et al.*: (1.3 ± 0.4) × 10^10^ s^−1^ and (1.5 ± 0.4) × 10^10^ s^−1^, respectively. The similarity of the predictions of the two models can also be seen from the three-dimensional representations of the anisotropic component of *Φ*_SS_ shown in figure 4*c*,*d* for three values of *f*_C_ with *k*_CD_ = 3 × 10^10^ s^−1^. Although the calculations shown in figure 4 included only two hyperfine interactions (FN5 and WN1), there is no reason to think that the composite model would be significantly less valid for radical pairs with a more realistic number of nuclear spins (see electronic supplementary material, section S1.4 for details).

Figure 4 confirms that if the RP1_C_ ↔ RP1_D_ interchange is fast enough, the composite model provides a reliable picture of the overall magnetic sensitivity of the system. This is a considerable simplification both conceptually and computationally and has allowed figure 5 to be calculated with three instead of two nuclear spins. This difference explains the less structured appearance of figure 5*b* compared to figure 4*a*,*b* (see electronic supplementary material, section S4).

With its validity confirmed, the composite model was then used to explore the dependence of the signal on the different degrees of freedom available to the system: the reaction rate constants, the spin-relaxation rate and the position of the equilibrium. Figure 5*a* shows contour plots of Δ*Φ*_SS_ calculated for weighted-average rate constants $\left\langle k_{r}\right\rangle \;\mathrm{and}\;\left\langle k_{f}\right\rangle$ in the range 10^4^−10^9^ s^−1^ (*y*- and *x*-axes, respectively; see electronic supplementary material, table S4) for seven values of *f*_C_ and three spin-relaxation rate constants. Note that these data are not presented in the same way as in figure 4 in which specific values of *k*_Cr_, *k*_Cf_, *k*_Dr_ and *k*_Df_ were used. By plotting Δ*Φ*_SS_ as a function of $\left\langle k_{r}\right\rangle \;\mathrm{and}\;\left\langle k_{f}\right\rangle$ in figure 5, two contour plots with the same value of *k*_relax_ (i.e. in the same row) and different values of *f*_C_ only differ in the weighted-average parameters of the TrpH^•+^ hyperfine and FAD^•−^–TrpH^•+^ dipolar interactions.

Within each contour plot, the maximum signal occurs for values of $\left\langle k_{r}\right\rangle \mathrm{and}\left\langle k_{f}\right\rangle$ near the centre of the 10^4^−10^9^ s^−1^ range, with $\left\langle k_{r}\right\rangle \approx 3\left\langle k_{f}\right\rangle$. This can be rationalized as follows [49]. If the recombination and forward reactions are too slow, the magnetic field effects are attenuated by spin relaxation. If they are too fast, there is insufficient time for the 50 µT magnetic field to affect the spin dynamics. If $\left\langle k_{r}\right\rangle \;\mathrm{and}\;\left\langle k_{f}\right\rangle$ are too different, the competition between the two reactions is ineffective.

Each column in figure 5*a* shows the effect of spin relaxation for a given value of *f*_C_. When the spins relax more rapidly, the signal strength drops and its maximum occurs for larger values of $\left\langle k_{r}\right\rangle \;\mathrm{and}\;\left\langle k_{f}\right\rangle$. The variations along the rows of figure 5*a* reflect the changes in the average dipolar and hyperfine interactions for different proportions of RP1_C_ and RP1_D_. Generally speaking, the signal is largest when 0.0 ≤ *f*_C_ ≤ 0.1 and drops as *f*_C_ increases. These variations can be seen more clearly in figure 5*b* for selected values of $\left\langle k_{r}\right\rangle \;\mathrm{and}\left\langle k_{f}\right\rangle$. They appear to result mainly from the dependence of the mean dipolar interaction on *f*_C_: the larger the dipolar interaction, the more it inhibits the singlet–triplet mixing caused by the magnetic field [44] (electronic supplementary material, section S4.3). Using centre-to-centre distances from FAD^•−^ to the two TrpH^•+^ radicals (electronic supplementary material, table S1), the average dipolar interaction rises from ⟨D⟩=−8.1 MHz (*f*_C_ = 0) to −14.3 MHz (*f*_C_ = 1).

The overall conclusion that can be drawn from figure 5 is that if there are no constraints on the values of the averaged rate constants $\left\langle k_{r}\right\rangle \;\mathrm{and}\left\langle k_{f}\right\rangle$, the largest signal available from the composite radical pair should occur for 0.0 ≤ *f*_C_ ≤ 0.1, i.e. ≥90% RP1_D_ rapidly interconverting with ≤ 10% RP1_C_.

## 5. Discussion

Three main conclusions come from the simulations presented in figures 4 and 5. (i) Provided their interconversion is fast enough (*k*_CD_ and *k*_DC_ > 10^10^ s^−1^), the third (RP1_C_) and fourth (RP1_D_) radical pairs formed by sequential electron transfers along the Trp-tetrad in *Er*Cry4a should behave as a single entity with weighted-average magnetic and kinetic properties (figure 4). (ii) If there are no restrictions on the values of the mean rate constants, $\left\langle k_{r}\right\rangle \;\mathrm{and}\left\langle k_{f}\right\rangle$, the largest anisotropic signals (Δ*Φ*_SS_) can be expected when the equilibrium proportion of RP1_D_ is 90–100% (i.e. *f*_C_ = 0.0–0.1, figure 5). (iii) The largest values of Δ*Φ*_SS_ (figure 5*a*) occur when $\left\langle k_{r}\right\rangle \approx 3\left\langle k_{f}\right\rangle$ and $\left\langle k_{f}\right\rangle \approx 2\;\times \;10^{6}\;s^{-1}$ (when *k*_relax_ = 10^5^ s^−1^), $\left\langle k_{f}\right\rangle \approx 6\;\times \;10^{6}\;s^{-1}$ (when *k*_relax_ = 10^6^ s^−1^) and $\left\langle k_{f}\right\rangle \approx 9\;\times \;10^{6}\;s^{-1}$ (when *k*_relax_ = 10^7^ s^−1^).

The immediate question raised by (ii) and (iii) is whether the values of $\left\langle k_{r}\right\rangle \;\mathrm{and}\left\langle k_{f}\right\rangle$ required to achieve large Δ*Φ*_SS_ are (i) realistic and (ii) compatible with small values of *f*_C_. The answer depends on the rate of spin relaxation (with which we start the discussion below). Before doing so, we note that Xu *et al*. [39] determined the strength of the dipolar interaction, *D*, in the FAD^•−^–TrpH^•+^ radical pair formed photochemically in WT *Er*Cry4a and hence the centre-to-centre separation of the radicals. Based on the crystal structure of the highly homologous pigeon protein, *Cl*Cry4a [37], the difference between the values of 〈*D*〉 expected for *f*_C_ = 0.0 and *f*_C_ = 0.1 was within the experimental error in the measurement of *D*. These experiments are, therefore, consistent with a small fraction of RP1_C_ (*f*_C_ ≤ 0.1) in rapid exchange with RP1_D_.

### 5.1. Spin-relaxation rates

As described in §3, there being no experimental measurements of spin-relaxation rates of radicals in Crys, the best estimates of *k*_relax_ come from molecular dynamics simulations combined with Bloch–Redfield relaxation theory which suggest values in excess of 10^6^ s^−1^ [45]. For the electrons to relax as slowly as 10^5^ s^−1^ the protein would either have to be almost rigid or the radicals within it would have to undergo very rapid, very low amplitude librational and torsional motions. Neither extreme is plausible.

A number of authors have used relaxation rates much slower than 10^6^ s^−1^ in computer simulations of magnetic field effects purporting to be relevant to magnetoreception. More commonly spin relaxation has been completely ignored. In our view, it is unrealistic to assume, in effect, that flavin and tryptophan radicals in a large protein behave in the same way as small radicals undergoing picosecond rotational diffusion in a non-viscous solvent, the only situation in which one could expect relaxation rates slower than about 10^6^ s^−1^ at physiological temperatures.

To summarize, the discussion below is based on the premise that *k*_relax_ ≥ 10^6^ s^−1^. This implies (figure 5*a*) that $\left\langle k_{r}\right\rangle \;\mathrm{and}\left\langle k_{f}\right\rangle$ must lie in the approximate range 10^6^–10^8^ s^−1^.

### 5.2. Recombination rates

We look first at the case of *f*_C_ = 0 in which RP1_D_ is solely responsible for the magnetic field effects. In this limit, the condition that $\left\langle k_{r}\right\rangle \approx \left\langle k_{f}\right\rangle$ ≥ 10^6^ s^−1^ is simply *k*_Dr_ ≈ *k*_Df_ ≥ 10^6^ s^−1^. A rough upper limit on the rate constant for direct back electron transfer from FAD^•−^ to Trp_D_H^•+^ (in s^−1^), assuming zero activation energy, can be obtained from [50]:

5.1 $$\log_{10}k_{r}\leq 13-0.6\;(R-3.6)$$

where *R* (in Å) is the edge-to-edge separation of the electron donor and acceptor. With *R* = 16.8 Å for RP1_D_ (electronic supplementary material, table S1), equation (5.1) gives *k*_Dr_ ≤ 1.1 × 10^5^ s^−1^. Using *R* = 16.0 Å and an approximate activation energy, Xu *et al*. [39] obtained a similar estimate: *k*_Dr_ ≈ (3.4 ± 1.5) × 10^5^ s^−1^. Such small values of $\left\langle k_{r}\right\rangle$ are not compatible with a large Δ*Φ*_SS_ when *k*_relax_ ≥ 10^6^ s^−1^. It is, therefore, difficult to see how RP1_D_ acting alone in *Er*Cry4a could form the basis of a sensitive magnetic compass. Müller *et al*. [51] reached the same conclusion based on measurements of electron transfer rates in *Xenopus laevis* (6–4) photolyase which also has a Trp-tetrad.

We now look at *f*_C_ = 0.1 to see whether a 1 : 9 combination of RP1_C_ and RP1_D_ in rapid equilibrium (figure 5) could make for a more satisfactory sensor. Applying equation (5.1) to RP1_C_, with an edge-to-edge separation of 13.6 Å (electronic supplementary material, table S1), one obtains an approximate upper limit on *k*_Cr_ of 1.1 × 10^7^ s^−1^. Xu *et al*. [39] arrived at the same number, *k*_Cr_ ≈ (1.2 ± 0.5) × 10^7^ s^−1^, using the slightly smaller separation of 13.3 Å and by including an activation energy term in equation (5.1). Combining *k*_Cr_ = 1.2 × 10^7^ s^−1^ with *k*_Dr_ = 1.1 × 10^5^ s^−1^ (from above) gives $\left\langle k_{r}\right\rangle \;=\;0.1k_{\mathrm{Cr}}\;+\;0.9k_{\mathrm{Dr}}\;\approx \;1.3\;\times \;10^{6}\;s^{-1}$ which satisfies one of the conditions for Δ*Φ*_ss_ to be relatively large, namely $\left\langle k_{r}\right\rangle \;\geq \;10^{6}\;s^{-1}$.

### 5.3. Tryptophan deprotonation rates

For a composite radical pair with $\left\langle k_{r}\right\rangle \;\approx \;1.3\;\times \;10^{6}\;s^{-1}$ and *f*_C_ = 0.1, to give a large value of Δ*Φ*_SS_, the mean rate constant for the forward reaction, $\left\langle k_{f}\right\rangle$, would (using the $\left\langle k_{r}\right\rangle \approx 3\left\langle k_{f}\right\rangle$ condition) need to be ≈ 4.3 × 10^5^ s^−1^. This reaction, in which the TrpH^•+^ radicals are stabilized by loss of the indole proton (WH1) to form neutral Trp^•^ radicals, has been studied for several members of the Cry-photolyase superfamily. Deprotonation time constants span four orders of magnitude, from 100–400 ps [52–54], to 200–400 ns [51,55–58], to 1–4 µs [41,51,59,60]. The very short, sub-nanosecond, lifetimes are for proteins that have an internal H^+^ acceptor and/or water molecules close to the indole nitrogen atom [52–54]; there is no evidence for either feature in the crystal structure of *Cl*Cry4a or in the molecular dynamics simulations of *Er*Cry4a for either Trp_C_H^•+^ or Trp_D_H^•+^ [39]. Slower deprotonation (greater than 100 ns) occurs when the solvent acts as the H^+^ acceptor. In only two cases have deprotonation rates been measured for Trp_C_H^•+^ and Trp_D_H^•+^ in the same protein (the former by replacing Trp_D_ by phenylalanine). Müller *et al*. [51] found time constants of 400 ns for Trp_C_H^•+^ and 2.5 µs for Trp_D_H^•+^ in *X. laevis* (6–4) photolyase, while Xu *et al*. [39] reported a 100 ns component in the decays of both RP1_C_ and RP1_D_ in *Er*Cry4a. The latter was interpreted in terms of a composite radical pair with $\left\langle k_{\mathrm{Cf}}\right\rangle \;\approx \;\left\langle k_{\mathrm{Df}}\right\rangle \;\approx \;(5-10)\times 10^{6}\;s^{-1}$.

While most of these measurements on purified proteins are inconsistent with $\left\langle k_{f}\right\rangle \;\approx \;4.3\;\times \;10^{5}\;s^{-1}$, there is no reason why release of the indole proton from either Trp_C_H^•+^ or Trp_D_H^•+^ *in vivo* necessarily occurs at the same rate as for the purified protein. Interactions of Crys with other proteins, required either for molecular alignment or signal transduction [42,61], could reduce the solvent accessibility and hence the deprotonation rate. Additionally, reasonably large values of Δ*Φ*_SS_ can still be expected (figure 5*a*) even though the optimum condition, $\left\langle k_{r}\right\rangle =3\left\langle k_{f}\right\rangle$, may not be satisfied exactly. In summary, it seems possible that $\left\langle k_{f}\right\rangle$, like $\left\langle k_{r}\right\rangle$, could, *in vivo*, fall in the range required for a large Δ*Φ*_SS_.

### 5.4. Tyr319 reduction

Potentially, there is an alternative reaction pathway from the composite radical pair to the SS. Tyr319 (figure 1) has an edge-to-edge distance to Trp_D_ of 3.9 Å (electronic supplementary material, table S1), and appears well placed to be oxidized by Trp_D_H^•+^. In several members of the photolyase-Cry superfamily there is a tyrosine at the far end of the Trp-triad that donates an electron to the terminal Trp_C_H^•+^ radical, thus extending the electron transfer chain and stabilizing the FAD^•−^ radical against back electron transfer [40,53,54,62–66]. The tyrosine radical (TyrO^•^) so formed is solvent-exposed and therefore able to be reduced by exogenous electron donors, potentially allowing the efficient formation of a SS containing FADH^•^ as the only radical.

**Figure 6.**
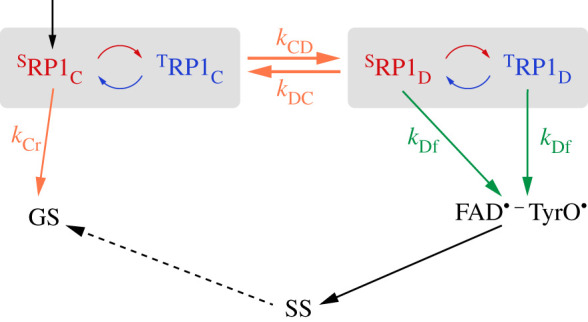
Modified version of figure 2*b* in which the major route to the SS is via reduction of Trp_D_H^•+^ by Tyr319 instead of deprotonation of Trp_C_H^•+^ and Trp_D_H^•+^. Contrary to a recent speculation [38], the edge-to-edge separation of FAD^•−^ and TyrO^•^ (20.3 Å, electronic supplementary material, table S1) is such that this radical pair is even less likely than RP1_D_ to recombine rapidly enough to give a significant magnetic field effect.

If Tyr319 oxidation rather than Trp_C,D_H^•+^ deprotonation is the major pathway to the SS, the reaction scheme in figure 2*b* changes to that shown in figure 6. Recombination may be taken to occur exclusively from RP1_C_ (smaller donor–acceptor separation than RP1_D_), while the SS is formed from RP1_D_ via spin-independent electron transfer from Tyr319 to Trp_D_H^•+^. Reduction of Trp_C_H^•+^ by Tyr319 is likely to be an order of magnitude slower given the approximately 3 Å larger donor–acceptor distance. Recombination of both RP1_D_ and [FAD^•−^ TyrO^•^] to the GS is assumed to be negligibly slow due to the large edge-to-edge distances, 16.8 and 20.3 Å, respectively (electronic supplementary material, table S1).

The conditions required for this modified reaction scheme (figure 6) to deliver large values of Δ*Φ*_SS_ are exactly the same as for the reactions in figure 2*b*, namely 10^6^ s^−1^ < $\left\langle k_{r}\right\rangle \approx 3\left\langle k_{f}\right\rangle$ < 10^8^ s^−1^ where the subscript in $\left\langle k_{f}\right\rangle$ now refers to electron transfer from Tyr319 to Trp_D_H^•+^. If, as assumed in figure 6, *k*_Cr_ ≫ *k*_Dr_ and *k*_Cf_ ≪ *k*_Df_, then $\left\langle k_{r}\right\rangle \approx f_{C}k_{\mathrm{Cr}}\;\mathrm{and}\left\langle k_{f}\right\rangle \approx (1-f_{C})k_{\mathrm{Df}}$. As long as *k*_Df_ is in the approximate range 10^6^−10^8^ s^−1^, the scheme in figure 6 could be just as suitable as that in figure 2*b* for efficient magnetic compass sensing.

There is some evidence that Tyr319 does indeed donate an electron to Trp_D_H^•+^ in avian Cry4a. Otsuka *et al*. [38] have reported long-lived TyrO^•^ radicals in chicken (*Gallus gallus*) Cry4a while Zoltowski *et al*. [37] found that mutating Tyr319 to aspartic acid in *Cl*Cry4a decreased the quantum yield of FAD photoreduction and modified the photoreduction kinetics. Xu *et al*. [39], however, did not detect light-induced TyrO^•^ radicals in robin Cry4a either by transient absorption or electron paramagnetic resonance, although a possible explanation in the former case is that the UV–visible absorption band of TyrO^•^ is narrow and heavily overlapped by the bands of the various FAD and Trp species. Nevertheless, it seems possible that Tyr319 could be the terminal electron donor in *Er*Cry4a *in vivo* where the rate of electron transfer to Trp_D_H^•+^ might be tuned by protein–protein interactions with signalling partners. If so, then reduction of Trp_D_H^•+^ by Tyr319 could, in conjunction with spin-selective recombination of RP1_C_, give rise to a significant Δ*Φ*_SS_.

An interesting feature of the scheme in figure 6 is that the two rate constants $\left\langle k_{r}\right\rangle =f_{C}k_{\mathrm{Cr}}$ and $\left\langle k_{f}\right\rangle =(1-f_{C})k_{\mathrm{Df}}$ depend on the properties of different tryptophan radicals (C and D, respectively), providing scope for independent optimization of $\left\langle k_{r}\right\rangle$ and $\left\langle k_{f}\right\rangle$. Amino acid mutations in the neighbourhood of Trp_C_H^•+^, for example, could tune $\left\langle k_{r}\right\rangle$ without affecting$\left\langle k_{f}\right\rangle$, and *vice versa*. This situation could also occur for the scheme in figure 2*b* if *k*_Cf_ ≪ *k*_Df_ and *k*_Cr_ ≫ k_Dr_ (see electronic supplementary material, section S2). By contrast, if only one radical pair is involved (figure 2*a*), a mutation that led to a favourable change in *k*_r_ might well have the opposite effect on *k*_f_.

## 6. Conclusion

By means of spin dynamics simulations, we have explored the potential advantages of simultaneously involving the third and fourth sequentially formed flavin–tryptophan radical pairs in magnetic sensing and signalling in avian Cry4a. A composite radical pair with weighted-average properties of its two components, could, at least in theory, offer ‘the best of both worlds'. That is, the stronger magnetic sensitivity afforded by [FAD^•−^ Trp_C_H^•+^] and the superior potential of [FAD^•−^ Trp_D_H^•+^] to form a SS via oxidation of Tyr319 (figure 6). Plants, whose Crys contain only three tryptophans, have no known biological requirement to respond to the direction of the Earth's magnetic field, and so might not need to separate the magnetic sensing and signalling functions in the same way as a migratory bird. The Cry from *Drosophila melanogaster* has four tryptophans, like avian Cry4a, but lacks the terminal tyrosine. This could be relevant if the various magnetic behaviours reported for fruit flies turn out to offer no biological advantage to these non-migratory animals [67–75].

Clearly, experiments are needed to test this idea. One possibility would be to mutate amino acid residues in the neighbourhood of the two tryptophans in such a way as to shift the position of the putative equilibrium. For example, introducing a negative charge or removing a positive charge in the vicinity of Trp_C_ could be expected to stabilize [FAD^•−^ Trp_C_H^•+^] and so change the magnetic sensitivity. Another option would be to seek conditions for the *in vitro* experiments that more closely resemble those *in vivo*. For example, it could be that electron transfer from Tyr319 to the fourth tryptophan radical is favoured by protein–protein interactions and could be revealed by studying Cry4a in association with one of the potential signalling partners identified by Wu *et al*. [42] Thus, it may be possible to get further insight into whether four tryptophans (or four tryptophans and a tyrosine) are better than three.

## Data accessibility

The data are provided in the electronic supplementary material [76].

## References

[1] Wiltschko R, Wiltschko W. 1995 Magnetic orientation in animals. Berlin, Germany: Springer.

[2] Mouritsen H. 2018 Long-distance navigation and magnetoreception in migratory animals. Nature **558**, 50-59. (10.1038/s41586-018-0176-1) 29875486

[3] Schulten K, Swenberg CE, Weller A. 1978 A biomagnetic sensory mechanism based on magnetic field modulated coherent electron spin motion. Z. Phys. Chem **111**, 1-5. (10.1524/zpch.1978.111.1.001)

[4] Ritz T, Adem S, Schulten K. 2000 A model for photoreceptor-based magnetoreception in birds. Biophys. J. **78**, 707-718. (10.1016/S0006-3495(00)76629-X) 10653784 PMC1300674

[5] Hore PJ, Mouritsen H. 2016 The radical pair mechanism of magnetoreception. Annu. Rev. Biophys. **45**, 299-344. (10.1146/annurev-biophys-032116-094545) 27216936

[6] Karki N, Vergisg S, Zoltowski BD. 2021 Cryptochromes: photochemical and structural insight into magnetoreception. Protein Sci. **30**, 1521-1534. (10.1002/pro.4124) 33993574 PMC8284579

[7] Wiltschko R, Niessner C, Wiltschko W. 2021 The magnetic compass of birds: the role of cryptochrome. Front. Physiol. **12**, 667000. (10.3389/fphys.2021.667000) 34093230 PMC8171495

[8] Wong SY, Frederiksen A, Hanic M, Schuhmann F, Grüning G, Hore PJ, Solovyov IA. 2021 Navigation of migratory songbirds: a quantum magnetic compass sensor. Neuroforum (10.1515/nf-2021-0005)

[9] Wiltschko W, Wiltschko R. 1972 Magnetic compass of European robins. Science **176**, 62-64. (10.1126/science.176.4030.62) 17784420

[10] Wiltschko R, Stapput K, Thalau P, Wiltschko W. 2010 Directional orientation of birds by the magnetic field under different light conditions. J. R. Soc. Interface **7**, S163-S177. (10.1098/rsif.2009.0367.focus) 19864263 PMC2843996

[11] Zapka M, Heyers D, Liedvogel M, Jarvis ED, Mouritsen H. 2010 Night-time neuronal activation of cluster N in a day- and night-migrating songbird. Eur. J. Neurosci. **32**, 619-624. (10.1111/j.1460-9568.2010.07311.x) 20618826 PMC2924469

[12] Zapka M et al. 2009 Visual but not trigeminal mediation of magnetic compass information in a migratory bird. Nature **461**, 1274-1278. (10.1038/nature08528) 19865170

[13] Chaves I et al. 2011 The cryptochromes: blue light photoreceptors in plants and animals. Annu. Rev. Plant Biol. **62**, 335-364. (10.1146/annurev-arplant-042110-103759) 21526969

[14] Liedvogel M, Mouritsen H. 2010 Cryptochromes—a potential magnetoreceptor: what do we know and what do we want to know? J. R. Soc. Interface **7**, S147-S162. (10.1098/rsif.2009.0411.focus) 19906675 PMC2844001

[15] Wang Q, Lin C. 2020 Mechanisms of cryptochrome-mediated photoresponses in plants. Annu. Rev. Plant Biol. **71**, 103-129. (10.1146/annurev-arplant-050718-100300) 32169020 PMC7428154

[16] Möller A, Sagasser S, Wiltschko W, Schierwater B. 2004 Retinal cryptochrome in a migratory passerine bird: a possible transducer for the avian magnetic compass. Naturwissenschaften **91**, 585-588. (10.1007/s00114-004-0578-9) 15551029

[17] Mouritsen H, Janssen-Bienhold U, Liedvogel M, Feenders G, Stalleicken J, Dirks P, Weiler R. 2004 Cryptochromes and neuronal-activity markers colocalize in the retina of migratory birds during magnetic orientation. Proc. Natl Acad. Sci. USA **101**, 14 294-14 299. (10.1073/pnas.0405968101) 15381765 PMC521149

[18] Niessner C, Denzau S, Gross JC, Peichl L, Bischof HJ, Fleissner G, Wiltschko W, Wiltschko R. 2011 Avian ultraviolet/violet cones identified as probable magnetoreceptors. PLoS ONE **6**, e20091. (10.1371/journal.pone.0020091) 21647441 PMC3102070

[19] Bolte P et al. 2016 Localisation of the putative magnetoreceptive protein cryptochrome 1b in the retinae of migratory birds and homing pigeons. PLoS ONE **11**, e0147819. (10.1371/journal.pone.0147819) 26953791 PMC4783096

[20] Niessner C, Gross JC, Denzau S, Peichl L, Fleissner G, Wiltschko W, Wiltschko R. 2016 Seasonally changing cryptochrome 1b expression in the retinal ganglion cells of a migrating passerine bird. PLoS ONE **11**, e0150377. (10.1371/journal.pone.0150377) 26953690 PMC4783048

[21] Günther A, Einwich A, Sjulstok E, Feederle R, Bolte P, Koch KW, Solovyov IA, Mouritsen H. 2018 Double-cone localization and seasonal expression pattern suggest a role in magnetoreception for European robin cryptochrome 4. Curr. Biol. **28**, 211-223. (10.1016/j.cub.2017.12.003) 29307554

[22] Einwich A, Dedek K, Seth PK, Laubinger S, Mouritsen H. 2020 A novel isoform of cryptochrome 4 (Cry4b) is expressed in the retina of a night-migratory songbird. Sci. Rep. **10**, 15794. 32978454 10.1038/s41598-020-72579-2 PMC7519125

[23] Hochstoeger T et al. 2020 The biophysical, molecular, and anatomical landscape of pigeon CRY4: a candidate light-based quantal magnetosensor. Sci. Adv. **6**, eabb9110. (10.1126/sciadv.abb9110) 32851187 PMC7423367

[24] Bolte P et al. 2021 Cryptochrome 1a localisation in light- and dark-adapted retinae of several migratory and non-migratory bird species: no signs of light-dependent activation. Ethol. Ecol. Evol. 33, 248-272. (10.1080/03949370.03942020.01870571)

[25] Pinzon-Rodriguez A, Muheim R. 2021 Cryptochrome expression in avian UV cones: revisiting the role of CRY1 as magnetoreceptor. Sci. Rep. **11**, 12683. (10.1038/s41598-021-92056-8) 34135416 PMC8209128

[26] Player TC, Hore PJ. 2019 Viability of superoxide-containing radical pairs as magnetoreceptors. J. Chem. Phys. **151**, 225101. (10.1063/1.5129608) 31837685

[27] Hogben HJ, Efimova O, Wagner-Rundell N, Timmel CR, Hore PJ. 2009 Possible involvement of superoxide and dioxygen with cryptochrome in avian magnetoreception: origin of Zeeman resonances observed by *in vivo* EPR spectroscopy. Chem. Phys. Lett. **480**, 118-122. (10.1016/j.cplett.2009.08.051)

[28] Pooam M, Arthaut LD, Burdick D, Link J, Martino CF, Ahmad M. 2019 Magnetic sensitivity mediated by the *Arabidopsis* blue-light receptor cryptochrome occurs during flavin reoxidation in the dark. Planta **249**, 319-332. (10.1007/s00425-018-3002-y) 30194534

[29] Niessner C, Denzau S, Peichl L, Wiltschko W, Wiltschko R. 2014 Magnetoreception in birds: I. Immunohistochemical studies concerning the cryptochrome cycle. J. Exp. Biol. **217**, 4221-4224. (10.1242/jeb.110965) 25472972 PMC4254396

[30] Wiltschko R, Ahmad M, Niessner C, Gehring D, Wiltschko W. 2016 Light-dependent magnetoreception in birds: the crucial step occurs in the dark. J. R. Soc. Interface **13**, 20151010. (10.1098/rsif.2015.1010) 27146685 PMC4892254

[31] Niessner C, Denzau S, Peichl L, Wiltschko W, Wiltschko R. 2018 Magnetoreception: activation of avian cryptochrome 1a in various light conditions. J. Comp. Physiol. A **204**, 977-984. (10.1007/s00359-018-1296-7) 30350127

[32] Müller P, Ahmad M. 2011 Light-activated cryptochrome reacts with molecular oxygen to form a flavin-superoxide radical pair consistent with magnetoreception. J. Biol. Chem. **286**, 21 033-21 040. (10.1074/jbc.M111.228940) PMC3122164 21467031

[33] Kutta RJ, Archipowa N, Johannissen LO, Jones AR, Scrutton NS. 2017 Vertebrate cryptochromes are vestigial flavoproteins. Sci. Rep. **7**, 44906. (10.1038/srep44906) 28317918 PMC5357904

[34] Ozturk N, Selby CP, Song SH, Ye R, Tan C, Kao YT, Zhong DP, Sancar A. 2009 Comparative photochemistry of animal type 1 and type 4 cryptochromes. Biochemistry **48**, 8585-8593. (10.1021/bi901043s) 19663499 PMC2739604

[35] Mitsui H, Maeda T, Yamaguchi C, Tsuji Y, Watari R, Kubo Y, Okano K, Okano T. 2015 Overexpression in yeast, photocycle, and *in vitro* structural change of an avian putative magnetoreceptor cryptochrome 4. Biochemistry **54**, 1908-1917. (10.1021/bi501441u) 25689419

[36] Wang X, Jing C, Selby CP, Chiou Y-Y, Yang Y, Wu WJ, Sancar A, Wang J. 2018 Comparative properties and functions of type 2 and type 4 pigeon cryptochromes. Cell. Mol. Life Sci. **75**, 4629-4641. (10.1007/s00018-018-2920-y) 30264181 PMC6383368

[37] Zoltowski BD et al. 2019 Chemical and structural analysis of a photoactive vertebrate cryptochrome from pigeon. Proc. Natl Acad. Sci. USA **116**, 19 449-19 457. (10.1073/pnas.1907875116) PMC6765304 31484780

[38] Otsuka H, Mitsui H, Miura K, Okano K, Imamoto Y, Okano T. 2020 Rapid oxidation following photoreduction in the avian cryptochrome 4 photocycle. Biochemistry **59**, 3615-3625. (10.1021/acs.biochem.0c00495) 32915550

[39] Xu J et al. 2021 Magnetic sensitivity of cryptochrome 4 from a migratory songbird. Nature **594**, 535-540. (10.1038/s41586-021-03618-9) 34163056

[40] Nohr D, Franz S, Rodriguez R, Paulus B, Essen L-O, Weber S, Schleicher E. 2016 Extended electron-transfer pathways in animal cryptochromes mediated by a tetrad of aromatic amino acids. Biophys. J. **111**, 301-311. (10.1016/j.bpj.2016.06.009) 27463133 PMC4968396

[41] Maeda K et al. 2012 Magnetically sensitive light-induced reactions in cryptochrome are consistent with its proposed role as a magnetoreceptor. Proc. Natl Acad. Sci. USA **109**, 4774-4779. (10.1073/pnas.1118959109) 22421133 PMC3323948

[42] Wu H, Scholten A, Einwich A, Mouritsen H, Koch KW. 2020 Protein–protein interaction of the putative magnetoreceptor cryptochrome 4 expressed in the avian retina. Sci. Rep. **10**, 7364. (10.1038/s41598-020-64429-y) 32355203 PMC7193638

[43] Haberkorn R. 1976 Density matrix description of spin-selective radical pair reactions. Mol. Phys. **32**, 1491-1493. (10.1080/00268977600102851)

[44] Babcock NS, Kattnig DR. 2020 Electron–electron dipolar interaction poses a challenge to the radical pair mechanism of magnetoreception. J. Phys. Chem. Lett. **11**, 2414-2421. (10.1021/acs.jpclett.0c00370) 32141754 PMC7145362

[45] Kattnig DR, Solovyov IA, Hore PJ. 2016 Electron spin relaxation in cryptochrome-based magnetoreception. Phys. Chem. Chem. Phys. **18**, 12 443-12 456. (10.1039/C5CP06731F) 27020113

[46] Kattnig DR, Sowa JK, Solov'yov IA, Hore PJ. 2016 Electron spin relaxation can enhance the performance of a cryptochrome-based magnetic compass sensor. New J. Phys. **18**, 063007. (10.1088/1367-2630/18/6/063007) 27020113

[47] Lee AA, Lau JCS, Hogben HJ, Biskup T, Kattnig DR, Hore PJ. 2014 Alternative radical pairs for cryptochrome-based magnetoreception. J. R. Soc. Interface **11**, 20131063. (10.1098/rsif.2013.1063) 24671932 PMC4006233

[48] Atkins C, Bajpai K, Rumball J, Kattnig DR. 2019 On the optimal relative orientation of radicals in the cryptochrome magnetic compass. J. Chem. Phys. **151**, 065103. (10.1063/1.5115445)

[49] Hore PJ. 2019 Upper bound on the biological effects of 50/60 Hz magnetic fields mediated by radical pairs. Elife **8**, e44179. (10.7554/eLife.44179) PMC6417859 30801245

[50] Moser CC, Anderson JLR, Dutton PL. 2010 Guidelines for tunneling in enzymes. Biochim Biophys Acta **1797**, 1573-1586. (10.1016/j.bbabio.2010.04.441) 20460101 PMC3509937

[51] Müller P, Yamamoto J, Martin R, Iwai S, Brettel K. 2015 Discovery and functional analysis of a 4th electron-transferring tryptophan conserved exclusively in animal cryptochromes and (6-4) photolyases. Chem. Commun. **51**, 15 502-15 505. (10.1039/C5CC06276D) 26355419

[52] Immeln D, Weigel A, Kottke T, Perez Lustres JL. 2012 Primary events in the blue light sensor plant cryptochrome: intraprotein electron and proton transfer revealed by femtosecond spectroscopy. J. Am. Chem. Soc. **134**,12 536-12 546. (10.1021/ja302121z) 22775505

[53] Muller P, Ignatz E, Kiontke S, Brettel K, Essen LO. 2018 Sub-nanosecond tryptophan radical deprotonation mediated by a protein-bound water cluster in class II DNA photolyases. Chem. Sci. **9**, 1200-1212. (10.1039/C7SC03969G) 29675165 PMC5885780

[54] Lacombat F, Espagne A, Dozova N, Plaza P, Ignatz E, Kiontke S, Essen LO. 2018 Delocalized hole transport coupled to sub-ns tryptophanyl deprotonation promotes photoreduction of class II photolyases. Phys. Chem. Chem. Phys. **20**, 25 446-25 457. (10.1039/C8CP04548H) 30272080

[55] Aubert C, Vos MH, Mathis P, Eker APM, Brettel K. 2000 Intraprotein radical transfer during photoactivation of DNA photolyase. Nature **405**, 586-590. (10.1038/35014644) 10850720

[56] Byrdin M, Sartor V, Eker APM, Vos MH, Aubert C, Brettel K, Mathis P. 2004 Intraprotein electron transfer and proton dynamics during photoactivation of DNA photolyase from *E. coli*: review and new insights from an ‘inverse’ deuterium isotope effect. Biochim. Biophys. Acta **1655**, 64-70. (10.1016/j.bbabio.2003.07.001) 15100018

[57] Byrdin M, Lukacs A, Thiagarajan V, Eker AP, Brettel K, Vos MH. 2010 Quantum yield measurements of short-lived photoactivation intermediates in DNA photolyase: toward a detailed understanding of the triple tryptophan electron transfer chain. J. Phys. Chem. A **114**, 3207-3214. (10.1021/jp9093589) 19954157

[58] Muller P, Bouly JP, Hitomi K, Balland V, Getzoff ED, Ritz T, Brettel K. 2014 ATP binding turns plant cryptochrome into an efficient natural photoswitch. Sci. Rep. **4**, 5175. (10.1038/srep05175) 24898692 PMC4046262

[59] Paulus B et al. 2015 Spectroscopic characterization of radicals and radical pairs in fruit fly cryptochrome-protonated and nonprotonated flavin radical-states. FEBS J. **282**, 3175-3189. (10.1111/febs.13299) 25879256

[60] Kutta RJ, Archipowa N, Scrutton NS. 2018 The sacrificial inactivation of the blue-light photosensor cryptochrome from *Drosophila melanogaster*. Phys. Chem. Chem. Phys. **20**, 28 767-28 776. (10.1039/C8CP04671A) PMC6250122 30417904

[61] Lau JCS, Wagner-Rundell N, Rodgers CT, Green NJB, Hore PJ. 2010 Effects of disorder and motion in a radical pair magnetoreceptor. J. R. Soc. Interface **7**, S257-S264. (10.1098/rsif.2009.0399.focus) 20007172 PMC2844003

[62] Aubert C, Mathis P, Eker APM, Brettel K. 1999 Intraprotein electron transfer between tyrosine and tryptophan in DNA photolyase from *Anacystis nidulans*. Proc. Natl Acad. Sci. USA **96**, 5423-5427. (10.1073/pnas.96.10.5423) 10318899 PMC21875

[63] Giovani B, Byrdin M, Ahmad M, Brettel K. 2003 Light-induced electron transfer in a cryptochrome blue-light photoreceptor. Nat. Struct. Biol. **10**, 489-490. (10.1038/nsb933) 12730688

[64] Oldemeyer S, Franz S, Wenzel S, Essen LO, Mittag M, Kottke T. 2016 Essential role of an unusually long-lived tyrosyl radical in the response to red light of the animal-like cryptochrome aCRY. J. Biol. Chem. **291**, 14 062-14 071. (10.1074/jbc.M116.726976) PMC4933166 27189948

[65] Oldemeyer S, Mittag M, Kottke T. 2019 Time-resolved infrared and visible spectroscopy on cryptochrome aCRY: basis for red light reception. Biophys. J. **117**, 490-499. (10.1016/j.bpj.2019.06.027) 31326107 PMC6697383

[66] Lacombat F, Espagne A, Dozova N, Plaza P, Muller P, Brettel K, Franz-Badur S, Essen LO. 2019 Ultrafast oxidation of a tyrosine by proton-coupled electron transfer promotes light activation of an animal-like cryptochrome. J. Am. Chem. Soc. **141**, 13 394-13 409. (10.1021/jacs.9b03680) 31368699

[67] Gegear RJ, Casselman A, Waddell S, Reppert SM. 2008 Cryptochrome mediates light-dependent magnetosensitivity in *Drosophila*. Nature **454**, 1014-1018. (10.1038/nature07183) 18641630 PMC2559964

[68] Gegear RJ, Foley LE, Casselman A, Reppert SM. 2010 Animal cryptochromes mediate magnetoreception by an unconventional photochemical mechanism. Nature **463**, 804-807. (10.1038/nature08719) 20098414 PMC2820607

[69] Foley LE, Gegear RJ, Reppert SM. 2011 Human cryptochrome exhibits light-dependent magnetosensitivity. Nat. Commun. **2**, 356. (10.1038/ncomms1364) 21694704 PMC3128388

[70] Yoshii T, Ahmad M, Helfrich-Forster C. 2009 Cryptochrome mediates light-dependent magnetosensitivity of *Drosophila*’s circadian clock. PLoS Biol. **7**, 813-819. (10.1371/journal.pbio.1000086) PMC2667543 19355790

[71] Fedele G, Green EW, Rosato E, Kyriacou CP. 2014 An electromagnetic field disrupts negative geotaxis in *Drosophila* via a CRY-dependent pathway. Nat. Commun. **5**, 4391. (10.1038/ncomms5391) 25019586 PMC4104433

[72] Fedele G et al. 2014 Genetic analysis of circadian responses to low frequency electromagnetic fields in *Drosophila melanogaster*. PLoS Genet. **10**, e1004804. (10.1371/journal.pgen.1004804) 25473952 PMC4256086

[73] Bae J-E, Bang S, Min S, Lee S-H, Kwon S-H, Lee Y, Lee Y-H, Chung J, Chae K-S. 2016 Positive geotactic behaviors induced by geomagnetic field in *Drosophila*. Mol. Brain **9**, 55. (10.1186/s13041-016-0235-1) 27192976 PMC4870802

[74] Marley R, Giachello CNG, Scrutton NS, Baines RA, Jones AR. 2014 Cryptochrome-dependent magnetic field effect on seizure response in *Drosophila* larvae. Sci. Rep. **4**, 5799. (10.1038/srep05799) 25052424 PMC4107376

[75] Giachello CNG, Scrutton NS, Jones AR, Baines RA. 2016 Magnetic fields modulate blue-light-dependent regulation of neuronal firing by cryptochrome. J. Neurosci. **36**, 10 742-10 749. (10.1523/JNEUROSCI.2140-16.2016) PMC5083005 27798129

[76] Wong SY, Wei Y, Mouritsen H, Solov'yov IA, Hore PJ. 2021 Cryptochrome magnetoreception: four tryptophans could be better than three. *FigShare*. 10.1098/rsif.2021.0601 PMC8580466 34753309

